# How the stimulus influences mind wandering in semantically rich task contexts

**DOI:** 10.1186/s41235-018-0129-0

**Published:** 2018-09-26

**Authors:** Myrthe Faber, Sidney K. D’Mello

**Affiliations:** 1 Department of Psychology, University of Notre Dame, Notre Dame, IN 46556 USA; 2 Donders Institute for Brain, Cognition and Behaviour, Centre for Cognitive Neuroimaging, Radboud University, Nijmegen, The Netherlands; 3 Department of Cognitive Neuroscience, Radboud University Medical Centre, Nijmegen, The Netherlands; 4 Institute of Cognitive Science, University of Colorado Boulder, Boulder, CO 80309 USA; 5 Department of Computer Science, University of Colorado Boulder, Boulder, CO 80309 USA

**Keywords:** Mind wandering, Attention, Memory, Comprehension

## Abstract

What do we think about when we mind wander and where do these thoughts come from? We tested the idea that semantically rich stimuli yield patterns of mind wandering that are closely coupled with the stimuli compared to being more internally triggered. We analyzed the content of 949 self-reported zone outs (1218 thoughts) and 519 of their triggers from 88 participants who read an instructional text and watched a film for 20 min each. We found that mind wandering associated with memory retrieval was more frequent than prospection and introspection across both stimuli. Over 70% of autobiographical and semantic memory retrievals were triggered by the content of the stimuli, compared to around 30% for prospective and introspective thoughts. Further, latent semantic analysis revealed that semantic and unspecific memories were more “semantically” similar to their triggers than prospective and introspective thoughts, suggesting that they arise from spontaneous associations with the stimulus. These findings suggest a re-evaluation of how internal concerns and the external world give rise to mind wandering and emphasize the importance of studying mind wandering in semantically rich contexts akin to much of the real world.

## Significance

Mind wandering frequently occurs during everyday activities such as reading a book or watching TV, but where do these thoughts come from and are they influenced by the ongoing activity? We analyzed the content of thoughts, their triggers, and thought trains during reading and film comprehension. We found that: (1) mind wandering associated with memory retrieval is highly common when processing semantically rich content; and (2) much of spontaneous thinking is driven by the stimulus itself. We propose that research should consider semantic information in the environment to better understand how internal concerns and the external world give rise to mind wandering.

## Background

When reading a text, listening to a conversation, or watching a film, some of our thoughts are focused on the content of the task at hand whereas others wander off towards past memories, introspections, prospections, and even fantasies. Experience sampling studies tell us that mind wandering is ubiquitous, occurring as much as 50% of the time in everyday life (Killingsworth & Gilbert, 2010). Although numerous studies have focused on analyzing the frequency of mind wandering across tasks and contexts (see Randall, Oswald, & Beier, 2014 for an overview), few have explored from where it arises and how it influences subsequent thinking. In fact, a recent review of mind wandering research identified “[characterizing] the environmental conditions and internal concerns that tend to initiate [it]” as a key issue (Smallwood & Schooler, 2015, p. 511). The emphasis on internal states poses an important challenge because much of psychological research focuses on establishing a relationship between a stimulus or manipulation and behavior, somewhat neglecting that much of our thinking is self-generated and often off-task. It is also possible that the task or stimulus itself is the driver of mind wandering, raising interesting questions about certain experimental effects (cf. Faber, Mills, Kopp, & D’Mello, 2017; Krasich et al., 2018). Here, we analyze the content of thoughts to provide insight into how task stimuli drive mind wandering.

We begin by considering the nature of mind wandering. The term itself captures one of its key characteristics: “wandering” meaning to “move hither and thither without fixed course or certain aim” (Christoff, Irving, Fox, Spreng, & Andrews-Hanna, 2016, p. 719). Indeed, mind wandering is less deliberate than goal-directed thinking, as its content mostly arises outside of cognitive control (Christoff et al., 2016). This is not to say that “undeliberate” thoughts are completely unconstrained – some spontaneous thoughts, such as rumination and obsessive thoughts, are strongly driven and automatically constrained by their affective salience (Christoff et al., 2016).

Importantly, sensory salience can lead to strongly constrained spontaneous remindings, for example, seeing a professor in a green tie and vest can automatically trigger memories about a pre-school teacher playing tricks on his or her students on St. Patrick’s Day (example from Ball & Little, 2006 p. 1173). In line with this, one study found that people mind wandered more about the past than the present or future while completing a vigilance task with emotionally valenced words (Plimpton, Patel, & Kvavilashvili, 2015), suggesting that emotional words trigger past memories. Similarly, another study (unexpectedly) found that when reading a text on membranes, people who had more experience with the topic of a text (e.g. years of formal biology education) mind wandered more about the past than about the future (Smallwood, Nind, & O’Connor, 2009). These findings suggest the content of mind wandering might be to some extent triggered by the stimulus, likely through activations of memory traces (Faber & Mills, 2018).

Of course, it is unlikely that all mind wandering arises from stimulus^1^ processing. People also frequently mind wander in situations that are devoid of much sensory or affective salience. Studies that have used semantically impoverished tasks, such as the Sustained Attention to Response (SART) task, have found a strong bias towards prospective mind wandering (e.g. Baird, Smallwood, & Schooler, 2011; McVay & Kane, 2013; Smallwood et al., 2009; Stawarczyk, Cassol, & D’Argembeau, 2013a; Stawarczyk, Majerus, Maj, Van der Linden, & D’Argembeau, 2011). According to the current concerns hypothesis (Klinger, 1978, 1999), personally relevant information, such as unfulfilled goals, is the source of much of spontaneous cognition. Indeed, when participants are asked to focus on their personal concerns or needs before engaging in a task, rates of mind wandering increase (Klinger, 2013; Kopp, D’Mello, & Mills, 2015a; Masicampo & Baumeister, 2011; Rummel & Nied, 2017; Stawarczyk, Majerus, & D’Argembeau, 2013b; Stawarczyk et al., 2011), suggesting that this type of mind wandering might to a large extent be driven by the importance of these current concerns rather than spontaneous associations elicited by the environment. This does not imply that prospective mind wandering is solely driven by internal concerns because a semantically rich environment might also provide cues that align with an individual’s active goals or concerns, triggering prospective thoughts (Klinger, 2013; McDaniel, Einstein, Guynn, & Breneiser, 2004; Stawarczyk et al., 2011).

Hence, to what extent is the content of mind wandering associated or triggered by the task stimulus versus more internally driven? We hypothesize that when people engage in semantically rich task contexts that give rise to spontaneous associations, such as reading or watching a film, we expect to find a greater propensity of mind wandering thoughts related to autobiographical and semantic memory retrieval compared to thoughts pertaining to more internally driven current concerns and feelings. We further hypothesize that the source of mind wandering varies systematically, such that autobiographical and semantic memories should be triggered by and align with the stimulus to a greater extent than prospective and introspective thoughts. This would suggest that the former are more likely to arise from stimulus processing whereas the latter are driven primarily (but not exclusively) by internal concerns of importance to the individual.

We leverage code and count techniques as well as computational linguistics approaches (latent semantic analysis [LSA]; Landauer, Folt, & Laham, 1998) to test these predictions when people engage in two real-world semantically rich activities: reading a text and watching a film. We asked 88 participants to report the verbatim content of their thoughts and what, if anything, triggered them whenever they caught themselves mind wandering during these 20-min long tasks. We then categorized the content of the reported thoughts using rubrics based on previous mind wandering (see below), diary, and mental time travel studies (e.g. Ball & Little, 2006; Berntsen & Hall, 2004; D’Argembeau, Renaud, & Van Der Linden, 2011; Hintzman, 2011; Kvavilashvili & Mandler, 2004; Mace, 2005; Miles & Berntsen, 2015; Schank, 1983; Seilman & Larsen, 1989). We compared the proportion of thoughts across categories, and for each category, computed the proportion of thoughts that were triggered by the stimulus, and importantly, whether the thought content was meaningfully related to the trigger.

Our study builds on previous studies that have compared the occurrence of task-related interferences and task-unrelated thoughts (e.g. Baird et al., 2011; Sarason, Sarason, Keefe, Hayes, & Shearin, 1986; Smallwood, Obonsawin, & Heim, 2003; Stawarczyk et al., 2011) and those that have distinguished among thoughts pertaining to sensory and emotional states, the self, current concerns, prospective memory, stimuli, environmental distractions, and fantasies (e.g. Baumeister, Vohs, & Oettingen, 2016; Krawietz, Tamplin, & Radvansky, 2012; Schooler, Reichle, & Halpern, 2004; Smallwood et al., 2016; Song & Wang, 2012). However, in most of these studies, participants did not report their thoughts verbatim (with the exception of Baird et al., 2011), but either rated them on a Likert-scale (e.g. using a 1–5 scale, to what extent a thought was related to a plan) or selected a thought category out of a number of options (e.g. “school-related,” “yourself,” “text-related,” “fantasies,” etc.; Krawietz et al., 2012), limiting an in-depth analysis of the thought content like in the present work.

We also collected data on people’s verbatim thought triggers and used LSA (Landauer et al., 1998) to test associations between the trigger and the subsequent mind wandering thought. For instance, the trigger “all the talk about water” from the text stimulus and the mind wandering memory-based thought, “[a] beach nearby me at home that I always go to,” are meaningfully related, because water and beach share associations like the sea and swimming. In contrast, the relationship between the prospective thought “what I am going to wear to class tomorrow” and its trigger “the red balloon” is less apparent, as clothes and balloons have less semantic overlap. The goal of this study is to tease apart these relationships to provide insight into how mind wandering emerges in semantically rich tasks contexts.

## Methods

### Participants

Participants were 88 undergraduate students from a medium-sized private U.S. university (*N* = 65) and a large public U.S. university (*N* = 23) who participated for course credit (69% female). Participants were on average 19 years old; 63% were Caucasian/White, 22% African-American/Black, 6% Hispanic, Latino, or of Mexican origin, 4% Asian, 4% American Indian or Native Alaskan, and 1% reported “other.” Because the primary goal of the study was to collect verbatim thought content, and our previous studies with similar stimuli used suggested that the number of self-caught mind wandering reports varied considerably across participants (Faber, Radvansky, & D’Mello, 2018; Kopp et al., 2015a; Kopp, Mills, & D’Mello, 2015b), we sought to collect as much data within the subject pool schedule. As such, we did not conduct an a priori power analysis.

### Ethics, consent, and permissions

Before the study, participants read and signed an agreement to participate and a (voluntary) data release form permitting the use of their data for publication. They were informed that they were free to withdraw at any time. All materials, procedures, and forms were approved by the Institutional Review Board for the Protection of Human Subjects at both universities.

### Materials

The text excerpt was taken from a book entitled *Soap-bubbles and the Forces which Mould Them* (Boys, 1890), which is an instructional text on a relatively unfamiliar topic (surface tension). The text is rich in semantic content as it describes a series of experiments that the reader has to visualize (e.g. “I have in my hand a common camel’s-hair brush. If you want to make the hairs cling together and come to a point, you wet it, and then you say the hairs cling together because the brush is wet”) (Boys, 1890, p. 15). To resemble naturalistic computerized reading, an average of 650 words were presented per screen resulting in ten screens of text. Participants read at their own pace and read on average 6.1 screens in the allotted 20 min. For the film, we used the first 20 min of the movie *Le Ballon Rouge* (‘The Red Balloon’), a 32.5-min French film with English subtitles about a young boy in Paris who finds a red balloon that follows him wherever he goes (Lamorisse, 1956).

### Procedure

All experimental procedures were delivered on a computer. Participants were informed that the primary task was to read/watch an excerpt from the book/film (order counterbalanced) for 20 min each. They were instructed to report mind wandering whenever they found themselves zoning out while completing the primary task. Participants received the following instructions:

*While you are [reading/ watching the film], you may find yourself thinking about something other than what you are [reading/watching]. This is called “zoning out.” We are interested in what types of things people think about during a task like this (and during other kinds of tasks). In order to examine this, if you catch yourself zoning out at any time during reading, simply press the key labeled “ZONE OUT” on the keyboard. Please locate the “ZONE OUT” key now.*

*When you indicate that you are zoning out the computer will ask you what you were* *just* *thinking about. It is perfectly normal to think about things that are not related to the task and to have different kinds of thoughts during different kinds of tasks. Please try your best to honestly assess your thoughts at the time when we ask.*

Whenever participants reported zoning out, they were further instructed as follows:

*In the space below please tell us what you were thinking about when you zoned out.*

*Was there something in the [text/video] that triggered this thought?*

*If yes then please describe what it was and if no then leave blank.*

The task paused while participants were reporting thoughts. Participants were informed that the reading and film comprehension phases of the study would last 20 min each and that reporting the content of their thoughts would not increase the amount of time in the study. They were encouraged to be as complete as possible when reporting their thoughts. After both phases of the study, participants were interviewed by the experimenter about whether the instructions were clear, what triggered their thoughts during both phases and whether they had any issues reporting thoughts.

## Results

### Number of thoughts and triggers

Participants reported a total of 949 instances of mind wandering (557 during text comprehension, 392 while watching the film; an average of 10.8 [*SD* = 6.08] per participant). Whereas most instances (77.1%) contained one mind wandering thought, 217 instances were associated with two or more thoughts (e.g. “[When I was Facetiming with my mom yesterday]_THOUGHT1_ [and] [step team tryouts]_THOUGHT2_”), resulting in a total of 1215 thoughts. We identified 43 thoughts that were explicitly aimed at gaining a deeper understanding of the text or film as on-task thoughts and excluded them (e.g. “The camel’s hair experiment performed in the text,” “Whether the man in the window was the one who wrote down something in the previous scene,” “How capillary action in plants carry water up the stem. I have an image in my head of water going up the stem of a plant”).

We obtained participants’ responses for 1082 thought triggers (we did not systematically obtain triggers for 136 thoughts from 10 participants due to experimental error). A total of 524 (48.4%) thoughts were accompanied with a trigger (302 in the text condition, 222 in the film condition). Upon closer inspection, 46 triggers contained content associated with the thought (e.g. “[The soap]_TRIGGER_ [reminded me that] [I need to give my dog a bath and make an appointment for him to get his nails clipped]_CONTENT_”). We separated the thought content from the trigger and counted it as a thought unless it fully overlapped with the reported thought. Eight triggers consisted of only thought content (e.g. “Fortunate to live in the country and have gone down to play by the brook”) and were therefore not counted as triggers but instead as thoughts. For three thoughts, no trigger was reported but the thought content explicitly mentioned the trigger. In those cases, we removed the trigger from the thought content and counted it as a trigger (e.g. “[Reading the name “Lear”]_TRIGGER_ [made me think of] [King Lear]_CONTENT_”). This resulted in a total of 1218 thoughts (732 from the text condition, 486 from the film condition; on average 13.8 mind wandering thoughts (*SD* = 8.04) per participant) and 519 triggers (298 in the text condition, 221 in the film condition). Out of these triggers, only 24 (4.58%) were related to internal states of the participants (e.g. “I’m just really bored”). All other triggers were related to the content of the stimuli.

### Thoughts per category

Two researchers—one of whom was naïve about the aims of the study— coded the following content categories: autobiographical memories; semantic memories; fantasies; prospection (including current concerns); task-related interferences; thoughts about the stimulus itself; environmental distractions; and introspection (see Appendix and Table 1 for the full rubric along with examples). When it was unclear whether a memory was semantic or autobiographical, it was categorized as an unspecific memory. Thoughts that did not clearly fit in a category were categorized as vague. The coders first coded 100 randomly selected thoughts and discussed their categorizations until they reached full agreement. They then independently coded the remaining instances, achieving fair agreement (Cohen’s κ = 0.71). Finally, the coders resolved all disagreements through discussion.

**Table 1:** Thought categories, frequencies, and examples

	Percent	*N*	Examples
Environmental distractions	2.79	34	“It is warm in this room,” “What are those red things in the monitor of the computer,” “A high pitch tone outside.”
Task-related interferences	8.21	100	“If the experimenters chose this text because it is very boring and they want us to zone out a lot,” “If I am going to be quizzed on this,” “I was wondering how much time was left.”
Stimulus	10.3	126	“I was thinking that the red balloon had to have been CGI or something,” “Is this really a book,” “How cute the little boy is.”
Semantic memories	13.7	167	“I thought about a scene from both “The Mummy” and “The Return of the Mummy”,” “A story about someone injuring their eyes when diving in with them open,” “Trying to remember which Millais painting had a bubble in it.”
Autobiographical memories	14.7	179	“I remember walking in lines like that in grade school,” “When I visited the Louvre and saw the Greek and Etruscan vases,” “Jumping into the lake last Saturday morning.”
Unspecific memories	4.68	57	“Friends potentially in a class together,” “My roommate playing in the marching band,” “My new swimsuit.”
Fantasies	3.12	38	“Imagining my friend asking me “so chem[istry] is your easiest class right?”,” “What would happen if there were some sort of emergency while I were taking this study,” “What if everyone all the time spoke out their thoughts out loud like I am typing right now. What would happen.”
Prospection	16.8	204	“Cheer practice tonight,” “School work that I have to complete,” “I need to activate my card.”
Introspection	16.9	206	“I am about to fall asleep,” “How much I hate math,” “I cannot believe how many random thoughts I have.”
Vague	8.78	107	“Sleeping,” “Water,” “Nothing in particular.”

Percentages represent percentage of all thoughts (total number of thoughts is 1218)

Table 1 gives an overview of percentages and number of thoughts per category. We used Wilcoxon’s signed rank test for paired samples at the participant level (non-parametric testing due to zero-inflated distributions and overdispersion; Bonferroni corrected for multiple comparisons; Table 2) to establish whether some categories were more frequent than others. Introspection, prospection, autobiographical and semantic memories, and thoughts about the stimulus and task occurred more frequently than fantasies and environmental distractions. Prospection and introspection were more frequent than task-related interferences, but not autobiographical and semantic memories. When we combined autobiographical, semantic, and unspecific memories into one memory category, we found that memories (*M* = 4.58, *SD* = 4.18) were significantly more frequent than prospection (*Z* = 4.22, *p* < 0.001) and introspection (*Z* = 4.10, *p* < 0.001). This suggests that the prospective bias observed in previous studies (e.g. Baird et al., 2011; McVay & Kane, 2013; Smallwood et al., 2009) might be limited to contexts relatively devoid of semantic content.

**Table 2:** Pairwise-comparisons between the frequencies of thought categories across participants using Wilcoxon’s signed rank test for paired samples

		Effect size *r =* $\frac{Z}{\surd \left({n}_x+{n}_y\right)}$ of the difference							
	Mean (SD)	Prospection	Autobiographical memory	Semantic memory	Stimulus	Task-related interferences	Unspecified memory	Fantasies	Environment
Introspection	2.34 (2.86)	0.062	0.056	0.069	0.193	0.284^*^	0.403^*^	0.484^*^	0.474^*^
Prospection	2.32 (2.25)		0.072	0.093	0.201	0.284^*^	0.430^*^	0.491^*^	0.469^*^
Autobiographical memory	2.03 (2.41)			0.029	0.111	0.226	0.355^*^	0.446^*^	0.385^*^
Semantic memory	1.90 (2.44)				0.107	0.178	0.331^*^	0.396^*^	0.372^*^
Stimulus	1.43 (1.95)					0.107	0.235	0.341^*^	0.334^*^
Task-related interferences	1.14 (1.98)						0.139	0.267^*^	0.274^*^
Unspecified memory	0.65 (0.92)							0.153	0.124
Fantasies	0.43 (0.89)								0.004
Environment	0.39 (0.79)								

Rows and columns are ordered by mean (greatest to smallest; top-bottom and left-right). ^*^ represents statistical significance at *α* = 0.001 (Bonferroni correction for 36 comparisons)

We conducted two follow-up analyses. First, we used thought-level mixed-effects logistic regressions to explore whether thoughts from each category (coded as 1 or 0) were more likely to occur when participants (added as a random intercept) completed the text or film comprehension task first. These analyses yielded no significant differences (all *p* values > 0.287). We therefore did not distinguish between the different orders in the subsequent analyses.

**Fig. 1:**
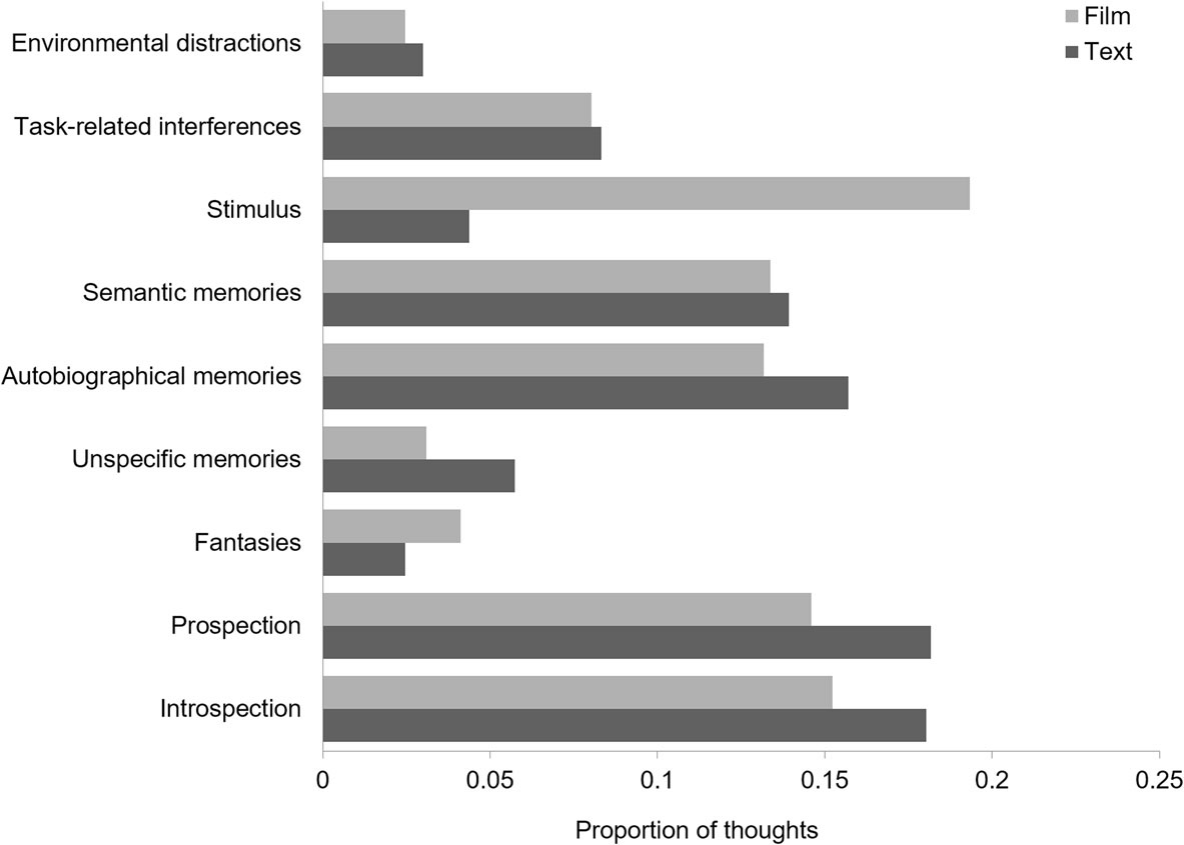
Proportions of thoughts per category per task pooled across thoughts (*light gray*: film comprehension, *dark gray*: text comprehension)

We also used thought-level mixed-effects logistic regressions to investigate whether thoughts from certain categories were more likely to occur during the text or film comprehension task (fixed effect) with participant as an intercept-only random effect. We found one significant difference (all other *p* values > 0.168) – thoughts about the stimulus were more likely during film comprehension (e.g. “Whether the balloon is real or picture animated,” “I wish this balloon made sound or something”) (odds ratio [OR] = 13.5, *SE* = 2.57, *p* = 0.006), which is unsurprising due to the comparatively stronger audiovisual information in the film compared to reading text (Fig. 1).

### Triggers per category

Where do mind wandering thoughts come from? It is clear that thoughts about external distractors, the task, and stimuli are cued by the environment. But what about thinking of “a cat I had that used to follow me around everywhere” or “what if the kitty of Alice in Wonderland is actually who is leading the balloon?” In particular, we asked whether memories were more likely to be triggered by the stimulus compared to introspective and prospective thoughts that are considered to arise primarily from feelings and current concerns, respectively. We used thought-level mixed-effect logistic regressions to predict whether a thought was triggered (1) or not (0) from the thought category, using participant as a random intercept. To test whether patterns were the same across reading and film comprehension, we added task as an interaction term. This led to convergence issues, so we repeated the analysis for each task separately. In a preliminary analysis, we ascertained that order (text or film comprehension first) did not affect the likelihood of a thought being triggered (OR = 0.867, *SE* = 1.37, *p* = 0.652), so we did not distinguish between orders here.

**Fig. 2:**
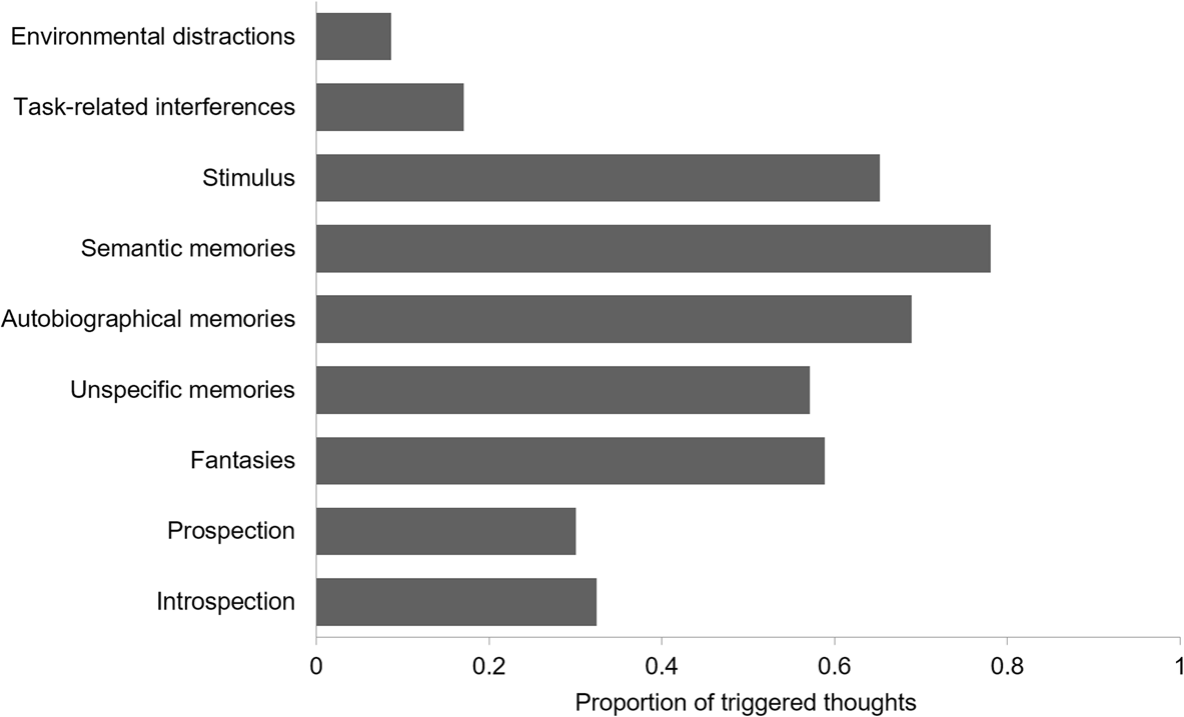
Proportion of triggered thoughts per category

We found that the likelihood of a thought being triggered differed across thought categories (main effects: Wald *χ*^*2*^ (9) = 90.2, *p* < 0.001 for text comprehension, Wald *χ*^*2*^ (4) = 47.9, *p* < 0.001 for film comprehension) (Fig. 2). Planned comparisons (estimated marginal means; Lenth, 2018) indicated that semantic memories were indeed more likely to be triggered than prospection (text/film: OR = 15.4/18.3, *SE* = 6.34/11.7, both *p* < 0.001) and introspection (text/film: OR = 13.6/14.6, *SE* = 5.64/8.92, both *p* < 0.001). We found the same pattern for autobiographical memories (text/film: OR = 8.85/12.3, *SE* = 3.31/7.67, both *p* < 0.001 for introspection; OR = 7.83/9.82, *SE* = 3.00/6.12, both *p* < 0.001 for prospection). Unspecific memories followed a similar pattern, but differences were only significant for the text condition, likely because there were only 11 unspecific memories for the film condition (text/film: OR = 5.38/5.93, *SE* = 2.61/6.07, *p* < 0.001/*p* = 0.082 for introspection; OR = 4.76/4.73, *SE* = 2.33/4.66, *p* = 0.002/ *p* = 0.115 for prospection). In separate analyses for each thought category, we ascertained that the likelihood of a thought being triggered did not vary as a function of task for any of these categories (all *p* values > 0.360). Together, these findings suggest that memories might arise from processing semantically rich information, whereas prospection and introspection might primarily (albeit not exclusively) be driven more by internal factors.

### Relationship between thoughts and triggers

We then asked how thought content is related to the reported triggers. We hypothesized that the relationship between thoughts and triggers should be stronger for thoughts associated with memory retrieval compared to thoughts that primarily arise from current concerns, such as prospection or introspection. We used an open source implementation (Olney, 2009) of LSA (Landauer et al., 1998) – a computational technique to measure the semantic similarity between two texts based on a reference semantic space – to obtain a measure of semantic overlap between each thought and its trigger. We used the Touchstone Applied Science Associates (TASA) corpus (with 300 dimensions with log entropy weighting) for the semantic space. We then used linear mixed-effects regression to test whether semantic overlap differed across content categories and tasks, controlling for the number of words in the thoughts and triggers. Participant identity was added to the model as a random intercept. We excluded thoughts pertaining to environmental distractors as there were insufficient instances for modeling (*N* = 2). In a separate analysis, we found that task order did not affect semantic overlap (Wald χ^2^ (1) = 0.008, *p* = 0.930) and was therefore not included in these analyses.

**Fig. 3:**
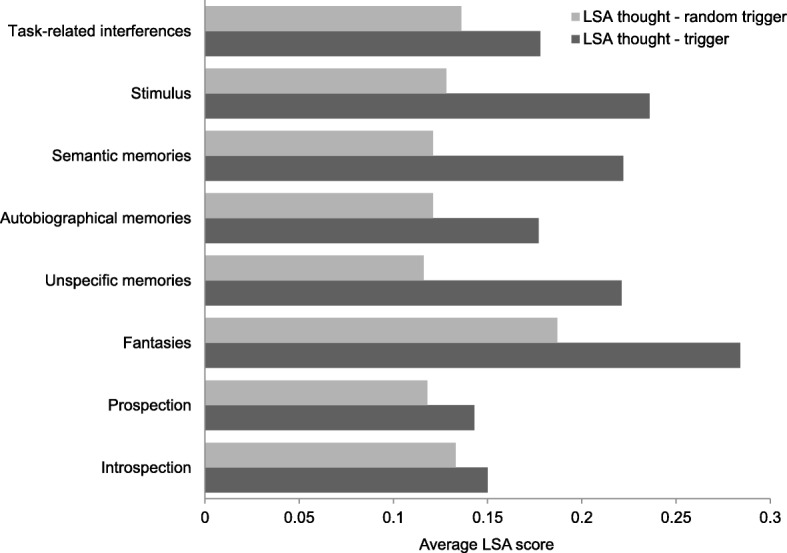
Average LSA score for the relationship between the thoughts and triggers (*dark gray*) and thoughts and random triggers (*light gray*) at the thought-trigger level

Semantic overlap between thoughts and triggers varied marginally across content categories (Wald *χ*^*2*^ (8) = 15.0, *p* = 0.059) and significantly across tasks (Wald *χ*^*2*^(1) = 4.20, *p* = 0.040) (Fig. 3). Overlap was on average higher for thoughts reported while watching the film (*M* = 0.225, *SD* = 0.203) than during text comprehension (*M* = 0.172, *SD* = 0.185). There was no interaction between task and content (Wald *χ*^*2*^ (8) = 10.3, *p* = 0.243). Planned comparisons between the memory-related categories, prospection, and introspection revealed that semantic memories were more similar to their triggers than prospective thoughts (*b* = 0.079, *SE* = 0.032, *p* = 0.014) and introspection (*b* = 0.069, *SE* = 0.031, *p* = 0.024). Unspecific memories displayed a similar pattern (*b* = 0.123, *SE* = 0.047, *p* = 0.010 compared to prospection and *b* = 0.113, *SE* = 0.046, *p* = 0.014 compared to introspection). Autobiographical memories did not differ significantly from either category (*b* = 0.039, *SE* = 0.032, *p* = 0.232 for prospection and *b* = 0.029, *SE* = 0.031, *p* = 0.353 for introspection). As an additional check, we confirmed that thoughts about the stimulus displayed a strong relationship with their triggers compared to thoughts about prospection and introspection (*b* = 0.084, *SE* = 0.036, *p* = 0.022 for prospection and *b* = 0.074, *SE* = 0.034, *p* = 0.031 for introspection).

If the content of thoughts is driven and constrained by the stimulus that triggered them, then breaking those links should lead to significantly weaker relationships. To test this hypothesis, we randomly shuffled the thoughts within each participant’s reports for a category to obtain a measure of overlap between a trigger and a randomly shuffled thought from the same category while accounting for the individual’s “thought space” (thoughts in that category for a given participant). Participants who only reported one trigger–thought pair for a category were excluded from the analysis for that category shuffling was not feasible. We tested whether LSA overlap differed significantly between the actual and the shuffled thoughts using the linear mixed-effects modeling approach above. We found that, as expected, the relationship between thoughts and shuffled triggers was significantly weaker for semantic, autobiographical, and unspecific memories (Wald *χ*^*2*^ (1) = 19.7, *p* < 0.001, Wald *χ*^*2*^ (1) = 5.92, *p* = 0.015, and Wald *χ*^*2*^ (1) = 5.91, *p* = 0.015, respectively) and for thoughts about the stimulus (Wald *χ*^*2*^ (1) = 29.4, *p* < 0.001) but not for introspection (Wald *χ*^*2*^ (1) = 1.07, *p* = 0.301), and prospection (Wald *χ*^*2*^ (1) = 0.311, *p* = 0.577). Thus, the results suggest that the relationship between thoughts and their triggers is meaningful for memory-related thoughts compared to introspective and prospective thoughts.

### Train of thoughts

If thoughts are driven by associations, then the content of one thought might trigger another (i.e. the experience of a *train of thought*) within the same mind wandering episode. For example, consider the following thought train from a participant: “beach nearby me at home that I always go to” → “my job as a beach tagger during high school” → “a guy that I used to like.” Or: “Louvre_TRIGGER_” → “the Louvre” → “haha last time I was in the Louvre I threw up in front of the Mona Lisa” → “I wonder how strange the people looking at this data will think I am” → “Maybe I should have admitted this after all.”

In our sample, 217 thoughts were followed by (at least) a second thought. For these instances, we computed the LSA score between the first and second thought; there were insufficient data to go to the third thought and beyond. We compared these scores to random surrogates obtained by pairing the same first thought with a second thought from a randomly selected episode from the same participant (e.g. the thought “beach nearby me at home that I always go to” would be paired with “my friend’s parents being here last weekend”). Participants who only reported one mind wandering episode were excluded from the analysis as thought pairs could not be shuffled. Using the linear mixed-effects modeling approach above (ignoring content category due to sample size), we found that the relationship between consecutive thoughts (*M* = 0.422, *SD* = 0.255) was stronger than between shuffled thoughts (*M* = 0.367, *SD* = 0.244); (Wald *χ*^*2*^ (1) = 4.89, *p* = 0.027). There was no significant interaction with task (*p* = 0.513), suggesting that the relationship between consecutive thoughts was stronger than between random thoughts for both reading and film comprehension. These findings suggest that the content of one thought can trigger another related thought to produce semantically related thought trains.

## Discussion

Our aim was to test the idea that the task stimulus itself might trigger certain types of mind wandering. We found that during real-world, semantically rich, reading and film comprehension tasks, memories (pooled across autobiographical, semantic, and unspecific memories) were almost twice as frequent as prospective (and also introspective) thoughts. Furthermore, approximately half of the mind wandering thoughts were triggered from the stimulus, a conservative estimate which relies on participants recalling the trigger (see below). Thoughts pertaining to memories were more likely to be triggered from the stimulus than prospective and introspective thoughts. Importantly the content of the semantic and unspecific memories was more strongly semantically related to their reported triggers than prospective and introspective thoughts, suggesting that the stimulus can drive and constrain the content of mind wandering that arises from memory associations.

The pattern of thought content observed here differs from many laboratory studies that have found that mind wandering thoughts tend to be focused on the future (e.g. Baird et al., 2011; McVay & Kane, 2013; Smallwood et al., 2009). The high prevalence of memories across both tasks, combined with the fact that we found no differences in the frequencies of prospective and memory-related thoughts across tasks, supports the idea that the content of mind wandering varies as a function of whether a task requires processing semantically rich information. Thus, the prospective bias observed in task contexts that are relatively devoid of semantic content might not generalize to real-world semantically rich tasks like those studied here, but would apply to other real-world tasks, such as vigilance tasks (Giambra, 1993).

Our findings also differ from experience sampling studies (e.g. asking people to report their thoughts throughout the day) which have suggested that much of mind wandering is future related (Song & Wang, 2012; Spronken, Holland, Figner, & Dijksterhuis, 2016). However, the relatively uncontrolled nature of these studies makes it difficult to investigate the relationship between what a person is doing and thinking as the data lack contextual detail and temporal precision. Based on our study, we would predict that mind wandering thoughts would more likely consist of memory-based retrievals when people engage in semantically richer activities like reading the newspaper or watching television, whereas prospection would be more frequent during more repetitive task like doing the dishes or vacuuming.

We do not claim that prospection is by definition stimulus-unrelated as around 30% of prospective thoughts were triggered by the stimulus content. This finding aligns with previous studies that have shown that cueing a person’s current concerns, for instance by asking them to make a to-do list (Kopp et al., 2015a) or read words that are related to the current concerns (McVay & Kane, 2013) can increase mind wandering. Examples from our data support this conclusion – we observed that “the passage [..] continuously talking about math” can trigger thoughts about “my math test at 11:20,” and seeing the letters “*AB* carved in the brick wall” can lead to “thinking about plans for the weekend” with a “friend [whose] initials are *AB*.” The current study suggests that stimulus processing can give rise to prospection if its content is related to the person’s concurrent goals. This also resonates with findings from the prospective memory literature, which suggest that cues that are related to a prospective memory (e.g. an unfulfilled task) may reflexively trigger spontaneous retrieval of that task or goal (McDaniel et al., 2004; Scullin, McDaniel, Shelton, & Lee, 2010).

A distinction based on whether a thought is directly triggered by the stimulus somewhat overlaps with the distinction between “stimulus-independent and task-unrelated thoughts” and “task-related interferences” (Frank, Nara, Zavagnin, Touron, & Kane, 2015; Stawarczyk et al., 2011; Zavagnin, Borella, & De Beni, 2014). However, our findings suggest that stimulus-dependence and task-relatedness are distinct dimensions in semantically rich task contexts. In particular, a task-unrelated thought (e.g. thinking about homework while reading a text) can be stimulus-dependent (e.g. triggered by the text) or stimulus-independent (e.g. arising from salient internal concerns). Similarly, task-related interferences can be more (e.g. wondering how long the passage of text would be) or less (e.g. wondering how many minutes have passed) stimulus-dependent. Although it might seem counterintuitive, thoughts can also be stimulus-independent, yet task-dependent. For example, reading a text on cell biology can lead one to deliberate on a previously studied genetics text – here the stimuli are different but the thought space is conceptually connected and such integration lies at the heart of deep learning (McNamara, Oreilly, & Vega, 2012).

The finding that mind wandering is to some extent driven by stimulus context prompts a definition that captures this quality. As we have shown here, defining mind wandering as “stimulus-unrelated thought” (Smallwood & Schooler, 2015) misses an important part of the phenomenon. Stimulus processing gives rise to mind wandering through spontaneous associations, which are relatively constrained by the semantic relationship with stimulus-based triggers. This is in line with the idea that mind wandering is a type of spontaneous thought that is relatively unconstrained by cognitive control (although some mind wandering might be intentional; see Seli, Risko, & Smilek, 2016), but varies in how strongly it is constrained by sensory (and affective) salience (Christoff et al., 2016).

We also observed that consecutive thoughts were more strongly semantically related than random thoughts sampled from an individual’s thought space, suggesting that the content of one thought triggers and constrains the next. It is also possible that another source (e.g. a potentially unreported thought or trigger) triggered both of them somewhat independently. Thoughts might also become more loosely associated over time, as one would expect during generation of new mental content (e.g. creative thinking) (Mills, Herrera-Bennett, Faber, & Christoff, 2018) but we could not analyze thought trains beyond the second thought due to a limited amount of data. Further research could shed light on how trains of thought unfold, elucidating the underlying principles of how the content of mind wandering arises.

It is important to consider some caveats with the present study. Because we focused on the self-caught method of reporting, mind wandering instances that did not reach meta-cognitive awareness might have been missed (Smallwood & Schooler, 2006). However, a benefit is that reports can occur at any time, independent of whether and when a participant received a thought probe, which is the more common way to track mind wandering (Giambra, 1995; Schooler et al., 2004). This is important for the purpose of the present study as it aims to elucidate the relationship between mind wandering and stimulus content without being limited to specific probe locations. An open question pertains to the systematic relationships between awareness of mind wandering, its content, and associated triggers and how the task context modulates these relationships. Although these aspects are beyond the scope of the present paper, establishing how internal and external triggers interact and compete to influence mind wandering and meta-cognitive awareness is an important step towards understanding the dynamics of spontaneous thought.

People also require awareness of the mind wandering triggers. Previous work has suggested that people might not actually be aware of how a stimulus influences their behavior but will still report a relationship when asked (Nisbett & Wilson, 1977), suggesting that people might infer a relationship based on causal theories or expectations. Therefore, it is possible that some triggers were inferred rather than remembered. That said, we aimed to avoid this by making reporting of the trigger voluntary. Specifically, we ask participants *whether* there was “something in the [text/video] that triggered this thought,” and explicitly gave instruction for both options (“If yes then please describe what it was and if no then leave blank”). The fact that participants reported triggers for around half of the thoughts suggests that they indeed did not feel compelled to report a trigger for every thought. Furthermore, the ~ 10% of the thought–trigger pairs for which thoughts and triggers were (inadvertently) reported together provide some insight into the validity of reported triggers. Examples suggest that these thoughts are triggered by a specific aspect of the stimuli, rather than post-hoc inference of the relationship: “The word “good” reminded me of my philosophy homework I haven’t finished,” “The boy was headed somewhere with his briefcase and it reminded me of what I have to do,” “I read the words “don’t know” and it reminded me of Socrates basically saying we don’t know wisdom, only God does.” Furthermore, there is also the possibility that it might be easier to remember and therefore report semantically associated triggers. These are known limits of verbal protocols and we are unaware of any alternative to obtain the contents of consciousness.

In addition, our self-caught approach ostensibly requires participants to divide attention between the primary task (reading text/watching film) and thought monitoring. It is possible that due to the demanding nature of the primary tasks, participants missed some instances of mind wandering. It is also possible that the extraneous load of simultaneous thought monitoring influenced how deeply participants processed the text or film. If processing was shallow, the frequency of associations triggered by the stimulus might be relatively low compared to when participants focus only on reading or watching the film. It might also be the case that constant thought monitoring resulted in an on average earlier termination of trains of thoughts. If the mind wanders further away from the stimulus as the train of thoughts continues, then our sample might be biased towards thoughts that are more closely related to the stimulus. Further research could shed light on these questions by exploring the relationship between thought content and the stimulus in a probe-caught or retrospective paradigm, although each has its limitations with regard to sampling frequency, probe placement, and the veracity of memories.

## Conclusions

In sum, our findings suggest that an analysis of mind wandering in semantically rich task contexts should account for multiple thought categories and associated triggers. Spontaneous associations that arise from stimulus processing are expected due to the associative nature of memory. These associations can be relevant to the task at hand and even enhance performance on the primary task as in the case of inference generation and creative ideation. However, as illustrated here, stimulus processing can also lead to retrieval of content that is irrelevant to the current task, such as memories, fantasies, and prospection. Importantly, the semantic-richness of the task context moderates (among other factors) the extent to which the stimulus activates different mind wandering thoughts. Semantically light environments should trigger a relatively high proportion of thoughts that arise from internal concerns, whereas semantically rich environments should trigger more stimulus-driven mind wandering. Whereas the present research has shown a higher propensity towards memory associations compared to thoughts arising from current concerns in semantically rich environments, further research is needed to make a more direct comparison between task contexts. We suggest that mind wandering research should move towards a comprehensive framework of when, why, and how the mind wanders when people engage in real-world tasks with varying degrees of semantic content.

## Figures and Tables

**Fig. 4:**
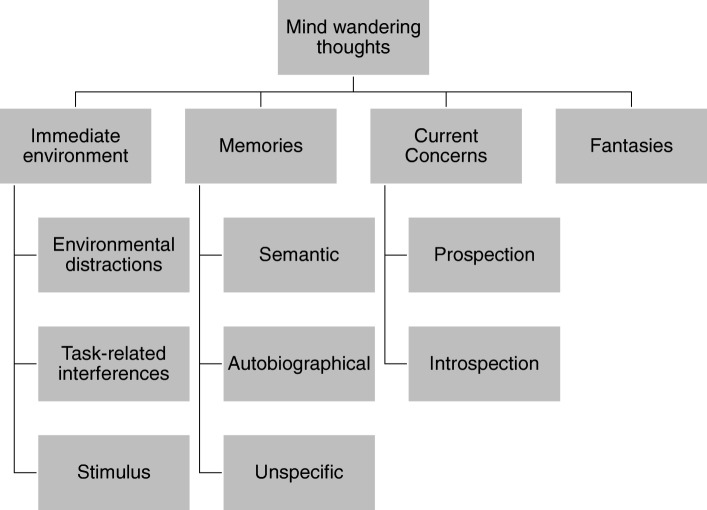
Overview of mind wandering content coding rubric

## Appendix

### Thoughts pertaining to the immediate environment

#### Environmental distractions

Thoughts pertaining to the immediate environment decoupled from the task. This includes thoughts about:

- Time, e.g. “I wonder what time it is.”
- Temperature and weather, e.g. “It is warm in this room,” “The weather is so gloomy.”
- Room, building, and items in it, e.g. “These eye sensors blink a lot,” “The fact that there are no windows in here.”
- External distractors, e.g. “Something making a faint sound near the desk,” “I hear bagpipes.”

#### Task-related interferences

Thoughts related to the task itself (rather than to the stimulus). This includes thoughts about:

- The experiment, e.g. “Why the experimenters would choose this movie,” “What zoning out really is and how I can tell if I’m doing it.”
- Progression of the experiment, e.g. “I was wondering what the next part of the study is,” “I wondered how much of the text I would have to read,” “Wondering how much time is left.”
- Apparatus, e.g. “The eye tracking device,” “What does this wrist band do.”

#### Stimulus

Thoughts related to the content or presentation of the text/film, but not associations going beyond the content. This includes thoughts about:

- The text/film content, e.g. “I wish this balloon made sound or sound or something,” “The name Simple Simon is ridiculous,” “The fakeness of the balloon,” “Whether the balloon is real or picture animated.”
- The text/film presentation, e.g. “These sentences are abnormally long,” “It is strange watching a video with this aspect ratio.”
- Thoughts, reflections, and inferences related to the immediate content of the film, e.g. “What is so special about this red balloon,” “I hope his balloon pops.”

### Thoughts pertaining to memories

#### Semantic memory

General knowledge (including facts, meaning, and concepts), e.g. “Math and physics formulae different equations math problems,” “Uptown Girls movie where the little girl gets a pet pig from her nanny,” “Song lyrics.”

#### Autobiographical memory

These are thoughts about events that are (1) related to the person themselves and (2) have actually happened, e.g. “When I rode on a bus in Colorado,” “Painting as a child.” They are anchored in time (and space). In some cases, the “when” can be inferred (e.g. “My high school bio class”).

#### Unspecific memory

Thoughts that require retrieval of information not immediately relevant to prospection/current concerns, but that do not meet the requirements for autobiographical memory, e.g. “My dog,” “Thinking about my grandpa.”

### Introspection

Thoughts related to meta-cognition or feelings or thoughts regarding the self. This includes thoughts about:

- Bodily feelings, e.g. “How tired I am,” “My knee itches,” “I am sleepy,” “Getting really hungry.”
- Emotional states, e.g. “Kind of confused,” “Frustrated,” “Worrying about my exams.”
- Mental states and reflections, e.g. “I miss my dog,” “It’s been a long week.”
- Reflections on task performance, e.g. “I am thinking about how difficult it is to focus on this page,” “I read really slow[ly],” “How I need to focus on reading,” “How much I zone out while studying,” “I’m not absorbing anything.”

### Prospection

Thoughts about the future, including current concerns. This includes thoughts about:

- Future plans, e.g. “My plans for the weekend,” “Assignments due tomorrow,” “Practice later,” “Where I’m going for dinner.”
- Prospective memory, e.g. “I need to put gas in my truck,” “Having to respond to a text telling work I did the laundry today,” “I need to get my nails done.”
- Planning, e.g. “Goals for the future,” “If I’m going to run and shower before [dinner] or wait until later and just run on the treadmill,” “Trying to make a plan of what I am going to do tonight.”
- Current concerns, e.g. “The word good reminded me of my philosophy homework I have not finished.”

### Fantasies

Fantasies and counterfactual situations, e.g. “What it would be like if there were a fire drill right now,” “I was imagining football players trying to stay on top of their school work,” “What would have happened if I had crossed the street and got hit. I would have had to tell someone I had Medex.”

### Vague

Thoughts that are none of the above. This includes thoughts about:

- No content, e.g. “I just started thinking,” “Nothing in particular,” “Do not remember.”
- Sleep, e.g. “Sleeping,” “A nap,” “Napping.”
- Unclear whether prospection or memory, or, e.g. “Traveling to India,” “Going to work,” “Soccer practice.”
- Unclear content, e.g. “Bese.”
- Too generic, e.g. “Lunch,” “Eating,” “Starbucks Coffee.”

### Not mind wandering

Thoughts that are immediately relevant to understanding the text or film: e.g. “How capillary action in plants carry water up the stem. I have an image in my head of water going up the stem of a plant,” “Hydrogen bonds and the chemical properties of water,” “Predicting a conclusion of water’s cohesive properties,” “How this has nothing to do with bubbles right now. Or maybe it does, but I can’t make the connection,” “Is letting go of the balloon and getting on the bus symbolic of growing? Of losing color in adulthood?”, “Is this balloon supposed to be a metaphor for something?”, “Trying to figure out the plot of this movie.” This includes wondering what a word means (e.g. “A water butt? I have never heard of that before,” “What’s a tumbler?”, “What duckweed looks like”) or what is on the screen (e.g. “I can’t tell what kind of animal that is”) and reflections on what was on the screen (e.g. “Yay! He got his balloon back!”, “Words!” [in response to the boy speaking in the movie]), as this is on-task behavior.

## Abbreviations

*LSA*: Latent semantic analysis

## Footnotes

1 We distinguish between the *content* of the task at hand (i.e. the stimulus) from the *context* in which it occurs (i.e. the environment). For example, if the task is to read a text in the lab, the stimulus is the text and the environment is the lab, the desk, etc.
